# Supplementary material for: Active site specificity profiling datasets of matrix metalloproteinases (MMPs) 1, 2, 3, 7, 8, 9, 12, 13 and 14
Source: Data Brief. 2016 Feb 22;7:299–310. doi: 10.1016/j.dib.2016.02.036 (PMC4777984; doi:10.1016/j.dib.2016.02.036)
Supplement: Supplementary file 10 — Supplementary material [file mmc10.zip › WebPICS_hMMP8_T_1%/PICS_results.html]

 

PICS results


- # About
- # Manual
- # Analysis
- # Results

- Redraw
- Seqlogo
- Dependency
- Coop

**PICS analysis of protease: hMMP8\_T\_1%**  
335 cleavage sites analyzed (total was 350)  
PICS library made with (T)rypsin, (G)luC or (C)hymotrypsin: T  

|  |  |
| --- | --- |
| Positional occurences    **Values > 10 % are shown** (table for total and relative (in %) values) | Occurences relative to natural abundance   **Values > 2 fold natural abundance are shown** (table) |
|  |  |

  
  
Results (including this html file) in one compressed file for local use.
  
  
Total occurences (open csv)

|  |  |  |  |  |  |  |  |  |  |  |  |  |
| --- | --- | --- | --- | --- | --- | --- | --- | --- | --- | --- | --- | --- |
|  | P6 | P5 | P4 | P3 | P2 | P1 | P1prime | P2prime | P3prime | P4prime | P5prime | P6prime |
| A | 12 | 22 | 14 | 44 | 25 | 35 | 4 | 17 | 74 | 48 | 27 | 25 |
| C | 9 | 2 | 3 | 7 | 11 | 9 | 17 | 5 | 1 | 2 | 3 | 3 |
| D | 17 | 13 | 18 | 9 | 28 | 29 | 0 | 3 | 23 | 21 | 28 | 25 |
| E | 22 | 13 | 26 | 15 | 29 | 43 | 0 | 7 | 14 | 27 | 26 | 31 |
| F | 5 | 15 | 11 | 4 | 23 | 15 | 25 | 17 | 13 | 9 | 17 | 9 |
| G | 18 | 22 | 21 | 15 | 10 | 30 | 2 | 4 | 75 | 25 | 22 | 36 |
| H | 6 | 8 | 10 | 5 | 7 | 7 | 3 | 12 | 11 | 8 | 12 | 11 |
| I | 8 | 9 | 19 | 19 | 7 | 4 | 52 | 35 | 8 | 20 | 28 | 19 |
| K | 1 | 0 | 3 | 0 | 0 | 0 | 0 | 7 | 1 | 13 | 5 | 10 |
| L | 22 | 33 | 26 | 27 | 51 | 15 | 130 | 60 | 4 | 33 | 36 | 28 |
| M | 5 | 5 | 10 | 6 | 13 | 6 | 16 | 21 | 2 | 13 | 5 | 5 |
| N | 9 | 11 | 12 | 5 | 12 | 41 | 5 | 5 | 13 | 9 | 13 | 12 |
| P | 16 | 16 | 14 | 68 | 2 | 16 | 1 | 0 | 4 | 23 | 27 | 30 |
| Q | 9 | 14 | 14 | 9 | 27 | 20 | 10 | 20 | 9 | 11 | 9 | 15 |
| R | 1 | 0 | 5 | 0 | 0 | 0 | 0 | 4 | 1 | 6 | 1 | 1 |
| S | 25 | 17 | 17 | 21 | 27 | 26 | 6 | 17 | 35 | 16 | 12 | 21 |
| T | 10 | 14 | 14 | 19 | 7 | 17 | 3 | 24 | 16 | 21 | 20 | 26 |
| V | 8 | 17 | 19 | 33 | 8 | 5 | 32 | 62 | 22 | 26 | 36 | 21 |
| W | 1 | 2 | 3 | 1 | 7 | 2 | 7 | 6 | 2 | 0 | 2 | 2 |
| Y | 11 | 6 | 17 | 5 | 24 | 3 | 22 | 9 | 7 | 4 | 6 | 5 |

  
  
Relative occurences in % (open csv)

|  |  |  |  |  |  |  |  |  |  |  |  |  |
| --- | --- | --- | --- | --- | --- | --- | --- | --- | --- | --- | --- | --- |
|  | P6 | P5 | P4 | P3 | P2 | P1 | P1prime | P2prime | P3prime | P4prime | P5prime | P6prime |
| A | 3.6 | 6.6 | 4.2 | 13.1 | 7.5 | 10.4 | 1.2 | 5.1 | 22.1 | 14.3 | 8.1 | 7.5 |
| C | 2.7 | 0.6 | 0.9 | 2.1 | 3.3 | 2.7 | 5.1 | 1.5 | 0.3 | 0.6 | 0.9 | 0.9 |
| D | 5.1 | 3.9 | 5.4 | 2.7 | 8.4 | 8.7 | 0.0 | 0.9 | 6.9 | 6.3 | 8.4 | 7.5 |
| E | 6.6 | 3.9 | 7.8 | 4.5 | 8.7 | 12.8 | 0.0 | 2.1 | 4.2 | 8.1 | 7.8 | 9.3 |
| F | 1.5 | 4.5 | 3.3 | 1.2 | 6.9 | 4.5 | 7.5 | 5.1 | 3.9 | 2.7 | 5.1 | 2.7 |
| G | 5.4 | 6.6 | 6.3 | 4.5 | 3.0 | 9.0 | 0.6 | 1.2 | 22.4 | 7.5 | 6.6 | 10.7 |
| H | 1.8 | 2.4 | 3.0 | 1.5 | 2.1 | 2.1 | 0.9 | 3.6 | 3.3 | 2.4 | 3.6 | 3.3 |
| I | 2.4 | 2.7 | 5.7 | 5.7 | 2.1 | 1.2 | 15.5 | 10.4 | 2.4 | 6.0 | 8.4 | 5.7 |
| K | 0.3 | 0.0 | 0.9 | 0.0 | 0.0 | 0.0 | 0.0 | 2.1 | 0.3 | 3.9 | 1.5 | 3.0 |
| L | 6.6 | 9.9 | 7.8 | 8.1 | 15.2 | 4.5 | 38.8 | 17.9 | 1.2 | 9.9 | 10.7 | 8.4 |
| M | 1.5 | 1.5 | 3.0 | 1.8 | 3.9 | 1.8 | 4.8 | 6.3 | 0.6 | 3.9 | 1.5 | 1.5 |
| N | 2.7 | 3.3 | 3.6 | 1.5 | 3.6 | 12.2 | 1.5 | 1.5 | 3.9 | 2.7 | 3.9 | 3.6 |
| P | 4.8 | 4.8 | 4.2 | 20.3 | 0.6 | 4.8 | 0.3 | 0.0 | 1.2 | 6.9 | 8.1 | 9.0 |
| Q | 2.7 | 4.2 | 4.2 | 2.7 | 8.1 | 6.0 | 3.0 | 6.0 | 2.7 | 3.3 | 2.7 | 4.5 |
| R | 0.3 | 0.0 | 1.5 | 0.0 | 0.0 | 0.0 | 0.0 | 1.2 | 0.3 | 1.8 | 0.3 | 0.3 |
| S | 7.5 | 5.1 | 5.1 | 6.3 | 8.1 | 7.8 | 1.8 | 5.1 | 10.4 | 4.8 | 3.6 | 6.3 |
| T | 3.0 | 4.2 | 4.2 | 5.7 | 2.1 | 5.1 | 0.9 | 7.2 | 4.8 | 6.3 | 6.0 | 7.8 |
| V | 2.4 | 5.1 | 5.7 | 9.9 | 2.4 | 1.5 | 9.6 | 18.5 | 6.6 | 7.8 | 10.7 | 6.3 |
| W | 0.3 | 0.6 | 0.9 | 0.3 | 2.1 | 0.6 | 2.1 | 1.8 | 0.6 | 0.0 | 0.6 | 0.6 |
| Y | 3.3 | 1.8 | 5.1 | 1.5 | 7.2 | 0.9 | 6.6 | 2.7 | 2.1 | 1.2 | 1.8 | 1.5 |

  
  
Occurences relative to natural abundance   

|  |  |  |  |  |  |  |  |  |  |  |  |  |
| --- | --- | --- | --- | --- | --- | --- | --- | --- | --- | --- | --- | --- |
|  | P6 | P5 | P4 | P3 | P2 | P1 | P1prime | P2prime | P3prime | P4prime | P5prime | P6prime |
| A | 0.4 | 0.8 | 0.5 | 1.6 | 0.9 | 1.3 | 0.1 | 0.6 | 2.7 | 1.7 | 1.0 | 0.9 |
| C | 2.0 | 0.4 | 0.7 | 1.5 | 2.4 | 2.0 | 3.8 | 1.1 | 0.2 | 0.4 | 0.7 | 0.7 |
| D | 0.9 | 0.7 | 1.0 | 0.5 | 1.5 | 1.6 | 0.0 | 0.2 | 1.3 | 1.2 | 1.5 | 1.4 |
| E | 1.0 | 0.6 | 1.2 | 0.7 | 1.3 | 1.9 | 0.0 | 0.3 | 0.6 | 1.2 | 1.2 | 1.4 |
| F | 0.4 | 1.2 | 0.9 | 0.3 | 1.8 | 1.2 | 1.9 | 1.3 | 1.0 | 0.7 | 1.3 | 0.7 |
| G | 0.8 | 0.9 | 0.9 | 0.6 | 0.4 | 1.3 | 0.1 | 0.2 | 3.2 | 1.1 | 0.9 | 1.5 |
| H | 0.8 | 1.1 | 1.3 | 0.7 | 0.9 | 0.9 | 0.4 | 1.6 | 1.5 | 1.1 | 1.6 | 1.5 |
| I | 0.4 | 0.5 | 1.0 | 1.0 | 0.4 | 0.2 | 2.6 | 1.7 | 0.4 | 1.0 | 1.4 | 1.0 |
| K | 0.1 | 0.0 | 0.2 | 0.0 | 0.0 | 0.0 | 0.0 | 0.4 | 0.1 | 0.7 | 0.3 | 0.5 |
| L | 0.7 | 1.0 | 0.8 | 0.8 | 1.6 | 0.5 | 4.0 | 1.9 | 0.1 | 1.0 | 1.1 | 0.9 |
| M | 0.6 | 0.6 | 1.2 | 0.7 | 1.6 | 0.7 | 2.0 | 2.6 | 0.2 | 1.6 | 0.6 | 0.6 |
| N | 0.7 | 0.8 | 0.9 | 0.4 | 0.9 | 3.0 | 0.4 | 0.4 | 1.0 | 0.7 | 1.0 | 0.9 |
| P | 1.0 | 1.0 | 0.9 | 4.3 | 0.1 | 1.0 | 0.1 | 0.0 | 0.3 | 1.5 | 1.7 | 1.9 |
| Q | 0.7 | 1.1 | 1.1 | 0.7 | 2.1 | 1.5 | 0.8 | 1.5 | 0.7 | 0.8 | 0.7 | 1.1 |
| R | 0.1 | 0.0 | 0.3 | 0.0 | 0.0 | 0.0 | 0.0 | 0.2 | 0.1 | 0.3 | 0.1 | 0.1 |
| S | 1.2 | 0.8 | 0.8 | 1.0 | 1.2 | 1.2 | 0.3 | 0.8 | 1.6 | 0.7 | 0.6 | 1.0 |
| T | 0.6 | 0.8 | 0.8 | 1.1 | 0.4 | 1.0 | 0.2 | 1.4 | 0.9 | 1.2 | 1.1 | 1.5 |
| V | 0.3 | 0.7 | 0.8 | 1.4 | 0.3 | 0.2 | 1.4 | 2.7 | 1.0 | 1.1 | 1.6 | 0.9 |
| W | 0.3 | 0.6 | 0.8 | 0.3 | 1.9 | 0.6 | 1.9 | 1.7 | 0.6 | 0.0 | 0.6 | 0.6 |
| Y | 1.1 | 0.6 | 1.8 | 0.5 | 2.5 | 0.3 | 2.3 | 0.9 | 0.7 | 0.4 | 0.6 | 0.5 |
